# Term Delivery in an Infertile Patient after Transcervical Radiofrequency Fibroid Ablation and Assisted Reproductive Technology

**DOI:** 10.1089/gyn.2019.0001

**Published:** 2019-08-01

**Authors:** Gregor Pschadka, Matthias Engelhardt, Caroline Niehoff, David Toub

**Affiliations:** ^1^ Department of Gynecology, Josephs-Hospital Warendorf, Warendorf, Germany.; ^2^ MVZ Kinderwunsch-und Hormonzentrum, Münster, Germany.; ^3^ Gynesonics, Redwood City, CA.

**Keywords:** uterine fibroids, radiofrequency ablation, Sonata System, assisted reproduction, pregnancy, transcervical

## Abstract

***Background:*** Transcervical radiofrequency (RF) ablation of uterine fibroids is an incisionless procedure to treat nonpedunculated uterine fibroids, including those that are not amenable to operative hysteroscopy. However, its safety and effectiveness regarding fertility and fecundity have not been established, including among women with infertility.

***Case:*** A 38-year-old nullipara with infertility since 2008 and a recent complaint of refractory dysmenorrhea in association with a uterine fibroid underwent transcervical RF ablation with the Sonata^®^ System (Gynesonics, Redwood City, CA) to treat the symptomatic myoma. Unsuccessful assisted reproduction (intracytoplasmic sperm injection/embryo transfer) as confirmed by negative pregnancy testing had been attempted 1 month preablation, and a second embryo transfer after thawing the previously cryopreserved pronuclei was performed 7 months postablation.

***Results:*** RF ablation with the Sonata System resulted in a 68% reduction in fibroid volume noted on sonography 2 months post-treatment along with resolution of the patient's dysmenorrhea. No residual fibroid was noted on sonography 7 months postablation. A second attempt at assisted reproduction produced an uncomplicated pregnancy that resulted in vacuum-assisted vaginal delivery of a liveborn infant at term weighing 3670 g with Apgar scores of 9^1^/10^5^/10^10^. Pelvic sonography 4 months postpartum an unremarkable uterus, again, with no evidence of a fibroid remnant.

***Conclusions:*** This is the first report of a pregnancy and delivery in an infertile couple who underwent transcervical RF ablation of a uterine fibroid followed by assisted reproduction.

## Introduction

Transcervical radiofrequency (RF) ablation is a technology that has demonstrated safety and efficacy for treating symptomatic uterine fibroids.^1–5^ While the clinical outcomes of RF ablation with regard to fertility and fecundity have not yet been definitively established, there are reports of pregnancies after both laparoscopic and transcervical RF ablations.^6–10^ In addition, a similar hyperthermic ablation technology, magnetic resonance–guided focused ultrasound (MRgFUS), has resulted in normal pregnancy outcomes and received an indication in women desiring future fertility by the U.S. Food and Drug Administration (FDA).

**FIG. 1.**
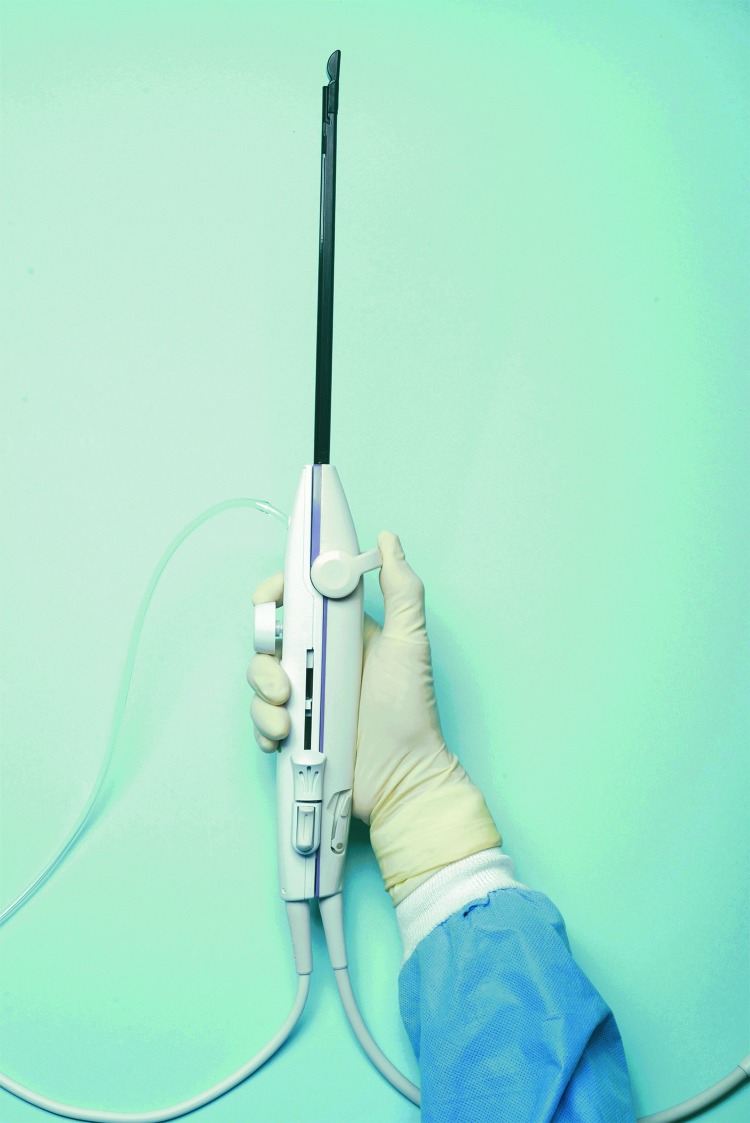
The Sonata^®^ System treatment device (Gynesonics, Redwood City, CA). Photo reproduced with permission.

The Sonata^®^ System (Gynesonics, Redwood City, CA; Fig. 1) is a device that provides transcervical RF ablation of uterine fibroids under real-time visualization provided by an integrated intrauterine ultrasound (US) probe.^5^ This device has received FDA clearance for diagnostic intrauterine imaging and transcervical treatment of symptomatic uterine fibroids, including those associated with heavy menstrual bleeding. The system also has a CE mark in the European Union. The Sonata System can be used to ablate nonpedunculated uterine fibroids, including those that are not amenable to operative hysteroscopy.^1,3,5^ Two separate prospective interventional trials of the Sonata System demonstrated significant reductions in menstrual bleeding and symptom severity, and improvements in health-related quality of life.^2,3^

There have been no reports of pregnancy after RF ablation of uterine fibroids in women who subsequently undergo assisted reproductive technology (ART) to manage infertility. This article describes the case of a patient who was treated with intrauterine US-guided transcervical RF ablation and was also managed with ART.

## Case

A 38-year-old gravida 1, para 0, abortus 1 patient presented in April 2017 with the chief complaint of dysmenorrhea over several months that had been refractory to medical therapy with nonsteroidal anti-inflammatory medications. Her past medical history was remarkable for hypothyroidism that was stable with medical management, a prior pregnancy that resulted in a spontaneous abortion, and a history of infertility since 2008. A reproductive workup revealed male-factor infertility. The patient had undergone her first ART cycle in March 2017; this consisted of intracytoplasmic sperm injection (ICSI) followed by embryo transfer (ET) but had not resulted in pregnancy. On sonographic examination, her uterus was 8 cm in length with a 3.6 cm × 3.2 cm × 3.2–cm fundal intramural myoma (volume 19.3 cc).

Upon being presented with her fibroid-treatment options, the patient chose transcervical RF ablation with the Sonata System, aware that its safety and effectiveness regarding future fertility was not established. After negative pregnancy testing, transcervical RF ablation was then performed in April 2017, 1 month after the couple's unsuccessful attempt at assisted reproduction. A single 3.4 cm × 3.8 cm ablation was made in the fibroid with the Sonata System under integrated, intrauterine sonographic guidance; there were no complications.

## Results

At a sonographic follow-up 2 months postablation, the fibroid was reduced in volume by 68%, measuring 2.5 cm × 2.1 cm × 2.2 cm (volume 6.1 cc), and the patient was free of dysmenorrhea. A second attempt at ART in November 2017 (7 months post-ablation), involving an ET after thawing of the previously cryopreserved pronuclei, did result in pregnancy. No residual fibroid was visible on a sonogram that documented an intrauterine gestation. After an unremarkable prenatal course, the patient underwent induction at 40 and 4/7 weeks' gestation, resulting in a liveborn 3670-g infant via operative vaginal delivery (vacuum assistance) with Apgar scores of 9^1^/10^5^/10^10^. There were no reported complications. Transvaginal sonography 4 months postpartum (20 months postablation) revealed a slightly smaller uterine length of 7.3 cm, again, with no visible fibroid remnant.

## Discussion

Uterine fibroids are highly prevalent and can produce significant symptoms in affected women, including heavy menstrual bleeding, pelvic pressure, and dysmenorrhea.^11–14^ While the most common treatment for fibroids is hysterectomy, transcervical RF ablation offers the potential for uterine conservation, significant symptom relief, a minimally invasive incisionless procedure, and brief treatment and recovery times. Surgical reintervention rates have been reported as ranging from 0.7% to 8% at 12 months and 11.8% at a mean of 64 months.^2–4^

Although there have been an increasing number of reports of successful pregnancies after RF ablation of uterine fibroids, none involved ART. There has been a report of successful ART after MRgFUS, which is similar to RF ablation in that it is a hyperthermic-ablation technology for treating uterine fibroids, but focused US is used rather than RF energy.^15^

The patient described herein underwent successful treatment of fibroid-associated dysmenorrhea with the Sonata System along with 2 cycles of ART involving ICSI and ET, resulting in a successful pregnancy delivered via vacuum extraction at term. Thus, this is the first reported case of an ART-related delivery after RF ablation of 1 or more fibroids.

It is not possible to determine conclusively if this patient's successful fibroid treatment was a significant factor in supporting her pregnancy. Any role of nonindenting fibroids in infertility remains to be determined, with some researchers finding no impact on fertility and others indicating a negative effect.^16–23^ With regard specifically to pregnancy after ART, there have been several reports (including a meta-analysis) that showed a deleterious impact of fibroids upon assisted reproduction, including ICSI.^20,21,23^ Thus, while speculative, it is possible that the resolution of the intramural fibroid after treatment with the Sonata System might have played a role in enabling successful ET postablation.

## Conclusions

This is the first report of pregnancy and vaginal delivery after transcervical ablation of a symptomatic uterine fibroid followed by ART. The normal antenatal and peripartum course following treatment with the Sonata System adds to the emerging literature base surrounding RF ablation and pregnancy, although such use in women who desire future fertility remains investigational.

## References

[1] Bongers M, Brölmann H, Gupta J, Garza-Leal JG, Toub D Transcervical, intrauterine ultrasound–guided radiofrequency ablation of uterine fibroids with the VizAblate^®^ System: Three- and six-month endpoint results from the FAST-EU study. Gynecol Surg 2015;12:61 25774122 10.1007/s10397-014-0873-1 PMC4349947

[2] Brölmann H, Bongers M, Garza-Leal JG, Gupta J, Veersema S, Quartero R, Toub D The FAST-EU trial: 12-month clinical outcomes of women after intrauterine sonography–guided transcervical radiofrequency ablation of uterine fibroids. Gynecol Surg 2016;13:27 26918001 10.1007/s10397-015-0915-3 PMC4753243

[3] Chudnoff S, Guido R, Roy K, Levine D, Mihalov L, Garza-Leal JG Ultrasound-guided transcervical ablation of uterine leiomyomas. Obstet Gynecol 2019;133:13 30531573 10.1097/AOG.0000000000003032

[4] Garza-Leal JG Long-term clinical outcomes of transcervical radiofrequency ablation of uterine fibroids: The VITALITY Study. J Gynecol Surg 2019;35:19 30713407 10.1089/gyn.2018.0051 PMC6354599

[5] Toub DB A New paradigm for uterine fibroid treatment: Transcervical, intrauterine sonography–guided radiofrequency ablation of uterine fibroids with the Sonata System. Curr Obstet Gynecol Rep 2017;6:67 28357157 10.1007/s13669-017-0194-2 PMC5350207

[6] Keltz J, Levie M, Chudnoff S Pregnancy outcomes after direct uterine myoma thermal ablation: Review of the literature. J Minim Invasive Gynecol 2017;24:538 28109894 10.1016/j.jmig.2017.01.009

[7] Berman JM, Bolnick JM, Pemueller RR, Garza Leal JG Reproductive outcomes in women following radiofrequency volumetric thermal ablation of symptomatic fibroids: A retrospective case series analysis. J Reprod Med 2015;60:194 26126303

[8] Berman JM, Puscheck EE, Diamond MP Full-term vaginal live birth after laparoscopic radiofrequency ablation of a large, symptomatic intramural fibroid: A case report. J Reprod Med 2012;57:159 22523877

[9] Bends R, Toub DB, Römer T Normal spontaneous vaginal delivery after transcervical radiofrequency ablation of uterine fibroids: A case report. Int J Womens Health 2018;10:367 30038526 10.2147/IJWH.S165959 PMC6052928

[10] Garza-Leal JG, León IH, Toub D Pregnancy after transcervical radiofrequency ablation guided by intrauterine sonography: Case report. Gynecol Surg 2014;11:145

[11] De La Cruz MS, Buchanan EM Uterine fibroids: Diagnosis and treatment. Am Fam Physician 2017;95:100 28084714

[12] Monleón J, Cañete ML, Caballero V et al.; EME Study Group. Epidemiology of uterine myomas and clinical practice in Spain: An observational study. Eur J Obstet Gynecol Reprod Biol 2018;226:59 29852335 10.1016/j.ejogrb.2018.05.026

[13] Foth D, Röhl FW, Friedrich C, et al. Symptoms of uterine myomas: Data of an epidemiological study in Germany. Arch Gynecol Obstet 2017;295:415 27873052 10.1007/s00404-016-4239-y

[14] David M, Pitz CM, Mihaylova A, Siedentopf F Myoma-associated pain frequency and intensity: A retrospective evaluation of 1548 myoma patients. Eur J Obstet Gynecol Reprod Biol 2016;199:137 26930041 10.1016/j.ejogrb.2016.02.026

[15] Zaher S, Lyons D, Regan L Successful *in vitro* fertilization pregnancy following magnetic resonance–guided focused ultrasound surgery for uterine fibroids. J Obstet Gynaecol Res 2011;37:370 21392163 10.1111/j.1447-0756.2010.01344.x

[16] Cook H, Ezzati M, Segars JH, McCarthy K The impact of uterine leiomyomas on reproductive outcomes. Minerva Ginecol 2010;62:225 20595947 PMC4120295

[17] Christopoulos G, Vlismas A, Salim R, Islam R, Trew G, Lavery S Fibroids that do not distort the uterine cavity and IVF success rates: An observational study using extensive matching criteria. BJOG 2017;124:615 27921379 10.1111/1471-0528.14362

[18] Sarıdoğan E, Sarıdoğan E Management of fibroids prior to *in vitro* fertilization/intracytoplasmic sperm injection: A pragmatic approach. J Turk Ger Gynecol Assoc 2018;20:55 30444214 10.4274/jtgga.galenos.2018.2018.0148 PMC6501871

[19] Somigliana E, De Benedictis S, Vercellini P et al. Fibroids not encroaching the endometrial cavity and IVF success rate: A prospective study. Hum Reprod 2011;26:834 21317415 10.1093/humrep/der015

[20] Vimercati A, Scioscia M, Lorusso F, et al. Do uterine fibroids affect IVF outcomes? Reprod Biomed Online 2007;15:686 18062866 10.1016/s1472-6483(10)60536-6

[21] Wang X, Chen L, Wang H, Li Q, Liu X, Qi H The impact of noncavity-distorting intramural fibroids on the efficacy of *in vitro* fertilization-embryo transfer: An updated meta-analysis. Biomed Res Int 2018;2018:8924703 30255100 10.1155/2018/8924703 PMC6142781

[22] Yan L, Ding L, Li C, Wang Y, Tang R, Chen ZJ Effect of fibroids not distorting the endometrial cavity on the outcome of *in vitro* fertilization treatment: A retrospective cohort study. Fertil Steril 2014;101:716 24424367 10.1016/j.fertnstert.2013.11.023

[23] Yan L, Yu Q, Zhang YN, Guo Z, Li Z, Niu J, Ma J Effect of type 3 intramural fibroids on *in vitro* fertilization-intracytoplasmic sperm injection outcomes: A retrospective cohort study. Fertil Steril 2018;109:817.e2 29605409 10.1016/j.fertnstert.2018.01.007

